# Multi-trait genetic analysis identifies novel pleiotropic loci for stroke and hematological traits or risk factors

**DOI:** 10.1016/j.fmre.2024.05.004

**Published:** 2024-05-20

**Authors:** Yue Jiang, Yingchao Song, Yaqi Li, Yuhao Tong, Huanxin Ding, Linzehao Li, Ziyue Yang, Ziang Meng, Miao Guo, Steven Weidong Su, Xiao Chang

**Affiliations:** a College of Artificial Intelligence and Big Data for Medical Sciences, Shandong First Medical University & Shandong Academy of Medical Sciences, Shandong 250117, China; b The First Affiliated Hospital of Shan Dong First Medical University, Shandong First Medical University & Shandong Academy of Medical Sciences, Shandong 250014, China; c Computer and Data Sciences, Case Western Reserve University, Cleveland, OH 44106, USA; d School of Life Sciences, Shandong First Medical University & Shandong Academy of Medical Sciences, Shandong 271016, China

**Keywords:** Stroke, Ischemic stroke, Hypertension, Venous thromboembolism, Genome-wide cross-trait analysis

## Abstract

Accumulating evidence suggests a correlation between stroke and hematological traits along with their associated risk factors, yet the precise shared genetic basis remains elusive. We collected GWAS summary statistics of stroke and potentially related hematological traits or risk factors from the GWAS Catalog and used LDSC to estimate genetic correlations and heritabilities. For trait pairs with significant correlations, we performed a comprehensive multi-trait GWAS analysis to identify pleiotropic loci shared by stroke and genetically correlated hematological traits or risk factors in both Europeans and East Asians. Our MTAG analysis identified 59 pleiotropic loci associated with both stroke and hypertension in Europeans, along with 17 such loci in East Asians. Among these loci, 33 were previously unknown in Europeans, and six were previously unknown in East Asians, suggesting potential new insights into the genetic basis of these traits. In the MTAG analysis of ischemic stroke and venous thromboembolism, we found six known loci and two novel loci. Notably, the 10q22.1 locus (rs12242391) was identified as an eQTL for the nearby gene TSPAN15 in multiple tissues, including whole blood. Furthermore, the 12q13.13 locus (rs4759076), which had not been reported in association with ischemic stroke or venous thromboembolism previously, exhibited a strong cis-eQTL effect with the nearby gene NFE2, a crucial regulator of megakaryocyte biogenesis and platelet production. Our study provided valuable insights into the shared genetic architecture of stroke and hematological traits or risk factors, highlighting potential avenues for future research and clinical applications.

## Graphical abstract

**Figure:**
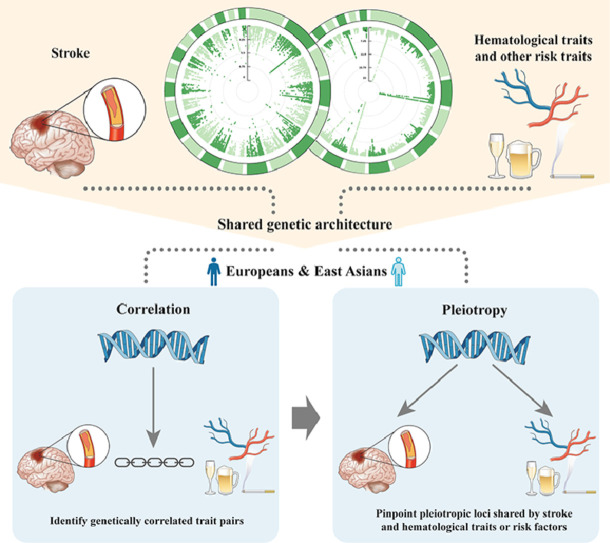

## 1 Introduction

Stroke is a cardiovascular disease (CVD) that occurs when the blood supply to a part of the brain is interrupted or reduced. According to the World Health Organization (WHO), stroke is the second leading cause of death and the third leading cause of disability-adjusted life-years (DALYs) worldwide [1]. The burden of stroke extends beyond mortality, as survivors often endure long-term impairments, including paralysis, cognitive deficits, and speech difficulties, imposing a heavy socioeconomic burden on individuals, families, and societies.

Epidemiological studies have identified several risk factors contributing to the occurrence of stroke, such as hypertension, smoking and drinking [[2], [3], [4]]. Besides, a growing body of evidence suggests that genetic factors play a crucial role in stroke susceptibility. The heritability of ischemic stroke has been estimated at 38%∼39%, varying across stroke subtypes [5,6]. To unravel the genetic underpins of stroke, multiple Genome-Wide Association Studies (GWAS) have been conducted. To date, a total of 89 significant loci have been detected in the largest GWAS of stroke comprising 110,182 cases and 1,503,898 controls [7], which indicates a polygenic model of inheritance. However, GWAS studies have primarily focused on individual traits in isolation, disregarding the potential shared genetic determinants among related phenotypes. Recently, multi-trait analysis of GWAS (MTAG) has emerged as a powerful approach to explore the genetic overlap and shared etiology among complex traits [8]. By jointly analyzing multiple genetic correlated traits, MTAG can identify pleiotropic genetic variants that influence related phenotypes, uncovering the genetic interplay between these conditions and the shared biological pathways implicated in their development.

The most significant recognized risk factor for stroke is hypertension, which is established as capable of causing the bursting or blockage of the arteries that deliver blood and oxygen to the brain [9]. Besides, certain hematological traits such as red blood cell distribution width and hemoglobin concentration have also been reported to be associated with stroke [10,11]. Hematologic abnormalities can also lead to thrombosis in the cerebral vasculature, causing ischemic cerebrovascular events. Although mechanisms of coagulation disorders that increase the risk of strokes are still not well understood, accumulating evidence suggests that coagulation factors may play a role in stroke pathophysiology [[12], [13], [14]]. In summary, stroke is likely influenced by a range of mechanisms linked to hematological risk factors, each with its own genetic background.

In this study, we first quantified genetic correlations between stroke and potentially related hematological traits or risk factors in Europeans and East Asians respectively. To further investigate the shared genetic architecture between the correlated traits, we conducted a comprehensive MTAG analysis and identified pleiotropic loci shared by stroke and genetic correlated hematological traits or risk factors in Europeans and East Asians.

## 2 Methods

### 2.1 GWAS data

The hitherto largest GWAS of stroke and subtypes (ischemic stroke, cardioembolic stroke, small vessel stroke and large artery stroke) was performed based on international collaborations in 110,182 cases and 1,503,898 controls [7]. GWAS summary statistics of stroke and subtypes from Europeans and East Asians were downloaded from GWAS Catalog [15]. Details of stroke GWAS datasets are presented in Table S1.

GWAS summary statistics of hematological traits or risk factors from Europeans including red blood cell (RBC) traits, platelet traits, blood pressure measurements, hypertension, protein levels of coagulation factors, venous thromboembolism and pulmonary embolism were also downloaded from GWAS Catalog [15]. For East Asian populations, only red blood cell (RBC) traits, platelet traits, blood pressure measurements and hypertension were available in GWAS Catalog [15]. Besides, GWAS summary statistics of smoking behavior and alcohol consumption were also analyzed. The information of all included GWAS data from Europeans and East Asians is summarized in Table S2.

The Institutional Review Board of Shandong First Medical University & Shandong Academy of Medical Sciences reviewed and approved the study. Given that the GWAS summary statistics data mentioned were sourced from the public domain, the board granted an exemption from requiring patient consent.

### 2.2 Global genetic correlation analysis

The cross-ancestry genetic correlation (R_g_) for stroke and subtypes between Europeans and East Asians was estimated using Popcorn [16], a computational method that determines the correlation of causal-variant effect sizes at SNPs common across population groups using GWAS summary-level data and LD information. Ancestry-specific LD scores were derived from the 1000 Genomes reference population [17]. The intra-ancestry genetic correlation R_g_ for stroke and subtypes, as well as for stroke and hematological traits was estimated by LD (Linkage Disequilibrium) score regression (LDSC) using GWAS summary statistics [18]. The LDSC method is described by the following equation: $E\left[\beta_{j}\gamma_{j}\right]=\frac{\sqrt{N_{1}N_{2}}r_{g}}{M}l_{j}+\frac{N_{s}r}{\sqrt{N_{1}N_{2}}}$, where $\beta_{j}$ and $\gamma_{j}$ denote the effect size of SNP *j* on the two tested traits, $N_{1}$ and $N_{2}$ are the sample sizes of two tested traits, $N_{s}$ is the number of overlapping samples between two tested traits, *r* is the phenotypic correlation in overlapping samples and $l_{j}$ is the LD score. Pre-computed linkage disequilibrium scores for HapMap3 SNPs calculated based on East-Asian-ancestry or European-ancestry individuals from the 1000 Genomes Project were used in the analysis, and SNP markers with an imputation INFO score < 0.9 were excluded. SNP based heritability of analyzed traits was also estimated by LDSC [18].

### 2.3 Cell-type-specific enrichment of SNP heritability

Stratified LDSC (s-LDSC) was used to detect potential functional categories or cell types contributing disproportionately to the heritability of studied traits. S-LDSC first divides the genome into non-overlapping sets of genomic regions based on various functional annotations or criteria, and then estimates the heritability for each subset or stratum of the genome. After estimating heritability for each stratum, s-LDSC compares the observed heritability in each stratum to the expected heritability based on the proportion of SNPs in that stratum. This comparison allows for the assessment of heritability enrichment within specific genomic annotations. Significant heritability enrichment in particular genomic annotations suggests that genetic variants within those regions play a disproportionate role in influencing the trait of interest. This information can provide insights into the biological mechanisms underlying the trait and prioritize genomic regions for further functional characterization.

In this study, annotation data were constructed by the Roadmap project for six chromatin marks (DHS, H3K27ac, H3K36me3, H3K4me1, H3K4me3, and H3K9ac) in a set of 88 cell types or tissues were used to partition the SNP heritability of each trait. For each chromatin mark, cell-type-specific annotations were further classified into nine broad groups including adipose, central nervous system, digestive system, cardiovascular, musculoskeletal and connective tissue, immune and blood, liver, pancreas, and others [19]. Annotation-specific enrichment values for each trait were transformed into color scale and visualized by hierarchical clustering.

### 2.4 Local genetic correlation analysis

Considering that genetic correlation estimated by LDSC aggregates information across all variants in the genome, we further estimated the pairwise local genetic correlation using ρ-HESS (heritability estimation from summary statistics) [20]. Ρ-HESS are designed to quantify the local genetic correlation between pairs of traits at each of the 1703 prespecified LD independent segments with an average length of 1.6 Mb. All significant trait pairs identified in the global genetic correlation analysis (LDSC, *p* < 0.01) underwent local genetic correlation analysis (ρ-HESS) to further investigate which specific local genomic regions contributed to the global genetic association. A Bonferroni corrected p value less than 0.05/1703 was considered as statistically significant.

### 2.5 Multi-trait analysis of GWAS

Multi-trait GWAS meta-analysis for stroke and hematological traits was performed by the Multi-Trait Analysis of GWAS (MTAG) framework, a generalized meta-analysis method that outputs trait-specific SNP associations [8]. MTAG can increase the power to detect loci from correlated traits by analyzing GWAS summary statistics jointly. It also accounts for sample overlap and incomplete genetic correlation, when comparing with the conventional inverse-variance weighted meta-analysis. The first step of MTAG is to filter variants by removing non common SNPs, duplicated SNPs, or SNPs with strand ambiguity. MTAG then estimates the pairwise genetic correlation between traits using LDSC [18] and uses these estimates to calibrate the variance-covariance matrix of the random effect component. MTAG next performs a random-effect meta-analysis to generate the SNP-level summary statistics. We prioritized significant pleiotropic SNPs that reached genome-wide significance (*p* < 5 × 10^–8^) in the multi-trait analysis and suggestive significance (*p* < 0.001) in the original single-trait GWAS.

### 2.6 Colocalization and gene-based analysis

Coloc [21] was used to investigate Bayesian colocalization of loci identified in MTAG analysis between stroke and related hematological traits or risk factors. With the default priors (p1 = 1  ×  10^−4^, p2 = 1  ×  10^−4^ and p12 = 1  ×  10^−5^), we considered evidence for colocalization when the posterior probability of shared causal variant (PP.H4) was greater than 0.8. Summary-data-based Mendelian Randomization (SMR) [22] was used to perform gene-based analysis. Each locus identified in MTAG underwent SMR analysis, using cis-eQTL summary data of whole blood from the GTEx v8. Within each locus, significant genes were identified using Bonferroni correction (corrected *p* < 0.05) to account for the multiple comparisons of tested genes. Additionally, genes exhibiting significant trends (*p* < 0.05) were also provided alongside the corrected results.

## 3 Results

### 3.1 Genetic correlations stroke and subtypes

To better understand the genetic architecture of stroke and its subtypes across different ancestries, we estimated the cross-ancestry genetic correlation between Europeans and East Asians using Popcorn. As shown in Table S3, there was no significant cross-ancestry genetic correlation for any stroke subtype. We also examined the genetic correlation between stroke and subtypes using LDSC in European and East Asian populations, respectively. In Europeans, all five subtypes of stroke exhibit significant positive correlation (Fig. S1 a), with the strongest associations observed between small vessel stroke and Stroke (R_g_ =1.22, *p* = 3.78 × 10^−16^), as well as between small vessel stroke and Ischemic stroke (R_g_ =1.15, *p* = 2.66 × 10^−17^). In East Asians, significant positive correlations exist among only four subtypes of stroke, including stroke, small vessel stroke, ischemic stroke, and large artery stroke. Notably, no significant association was found between cardioembolic stroke and any other stroke type (Fig. S1 b).

### 3.2 Genetic correlations between stroke and hematological traits or risk factors

**Fig. 1:**
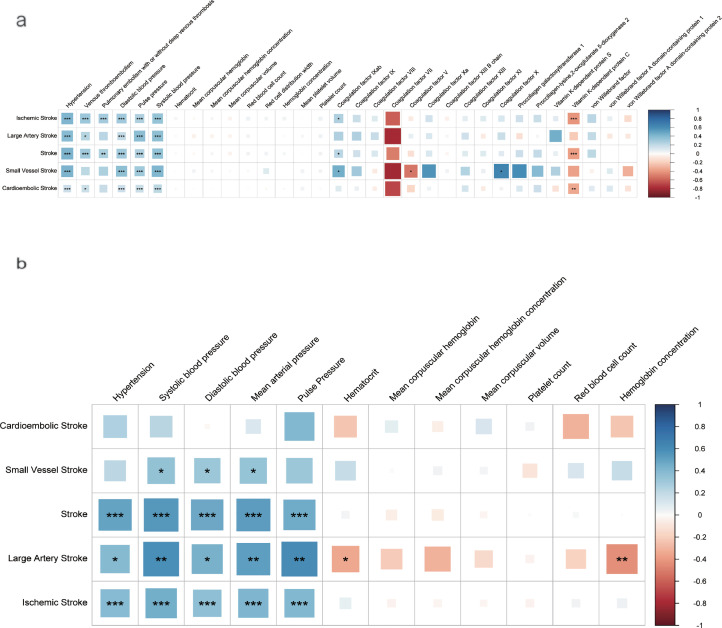
**Heatmaps of pairwise genome-wide genetic correlations between stroke and hematological traits in Europeans (a) and East Asians (b)**. The matrix displays the strength of correlation (size of each square) and significant associations are marked with an asterisk (*p* < 0.05), and the shade of each square denotes a positive (blue) or negative (red) correlation, varying in shade by magnitude of correlation.

We utilized LDSC to estimate the SNP-based heritability, which allowed us to determine the proportion of phenotypic variance explained by the tested variants. Heritability estimates on the observed scale using GWAS summary statistics of stroke is 0.59% and 1.5% (Table S4) in Europeans and East Asians. By comparison, hypertension demonstrated higher heritability estimates, with rates of 11% among Europeans and 7.7% among East Asians (Table S4). We next investigated genetic correlations between stroke and hematological traits or risk factors. With more GWAS data available for Europeans, we conducted a more comprehensive analysis, encompassing a broader range of risk factors. In Europeans, stroke and subtypes were significantly correlated with hypertension (R_g_ = 0.42, *p* = 2.2 × 10^−42^) and blood pressure measurements including diastolic blood pressure (DBP), pulse pressure and systolic blood pressure (SBP) (Fig. 1a and Table S5). Venous thromoboembolism and pulmonary embolism were also significantly correlated with stroke and ischemic stroke (Table S5). Whereas, a significant negative genetic correlation was detected between stroke and vitamin K-dependent protein C (PROC, R_g_ = −0.39, *p* = 3 × 10^−4^), which serves as the key component of an important natural anticoagulant pathway [23]. In contrast, no correlation was detected between stroke and red blood cell traits, or between stroke and platelet traits. In East Asians, stroke and subtypes were also significantly correlated with hypertension and blood pressure measurements (Fig. 1b and Table S5). Interestingly, large artery stroke was negatively correlated with hemoglobin concentration (R_g_ = −0.43, *p* = 9.3 × 10^−3^), which was not observed in Europeans.

### 3.3 Cell-type-specific enrichment of SNP heritability

For stroke and its subtypes, we further partitioned SNP heritability based on six chromatin marks and nine cell types. We found that both Europeans and East Asians exhibited similar patterns at six chromatin marks (Fig. S2) in hypertension, which differ from stroke and its subtypes, reflecting their unique genetic characteristics. Notably, tissues or cell types classified under the cardiovascular category, such as heart, atrium, ventricle and aorta, showed significant enrichment for stroke or its subtypes in chromatin marks H3K27ac, H3K4me3, and H3K9ac. We also observed that stroke and its subtypes exhibited significant heritability enrichments in tissues or cell types classified as musculoskeletal/connective tissue and digestive.

### 3.4 Local genetic correlations between stroke and hematological traits or risk factors

**Fig. 2:**
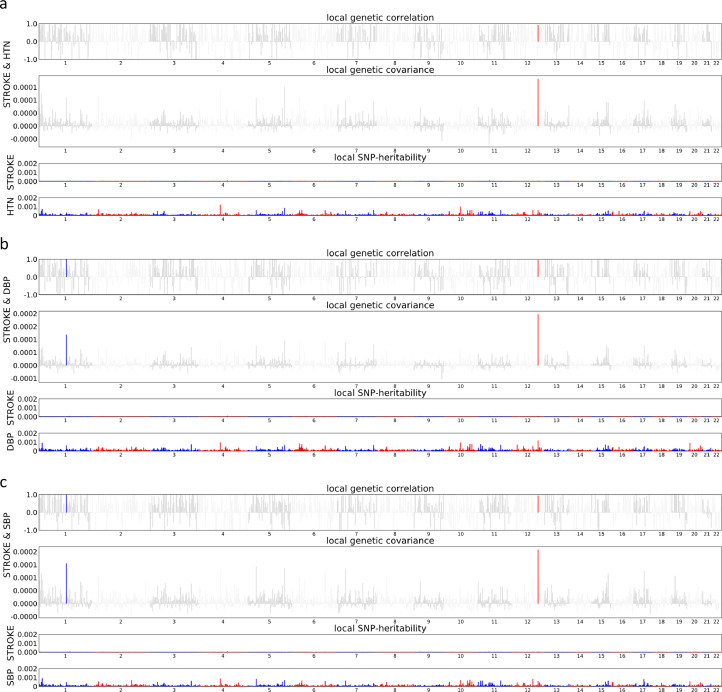
**Local genetic correlation between stroke and blood pressure measurements in Europeans.** (a) Manhattan plot showing the estimates of local genetic correlation, genetic covariance, and SNP heritability between stroke and hypertension in Europeans. (b) Manhattan plot showing the estimates of local genetic correlation, genetic covariance, and SNP heritability between stroke and diastolic blood pressure (DBP) in Europeans. (c) Manhattan plot showing the estimates of local genetic correlation, genetic covariance, and SNP heritability between stroke and systolic blood pressure (SBP) in Europeans. Red bars represent loci showing significant local genetic correlation after multiple testing adjustment (*p* < 0.05/1703).

We next conducted a comprehensive genome-wide scan to identify specific genomic regions that contribute to the shared heritability of genetically correlated traits. After correcting for multiple testing, we discovered a significant correlation between stroke and hypertension in Europeans, specifically at the 12q24 region (Fig. 2a). This finding was consistent with the observed significance of the 12q24 region in relation to stroke and various blood pressure measurements, such as DBP (Fig. 2b) and SBP (Fig. 2c). While no significant region was identified between stroke and hypertension in East Asians (Fig. S3a), we found that 12q24 displayed a correlation between stroke and blood pressure measurements, mirroring the findings in Europeans (Fig. S3b&c). Interestingly, previous studies have also identified significant GWAS signals in the 12q24 region for stroke, hypertension, and blood pressure measurements [7,24,25]. We did not find any significant correlated regions between stroke and other hematological traits or risk factors.

### 3.5 Multi-trait meta-analysis of stroke and hematological risk factors

**Fig. 3:**
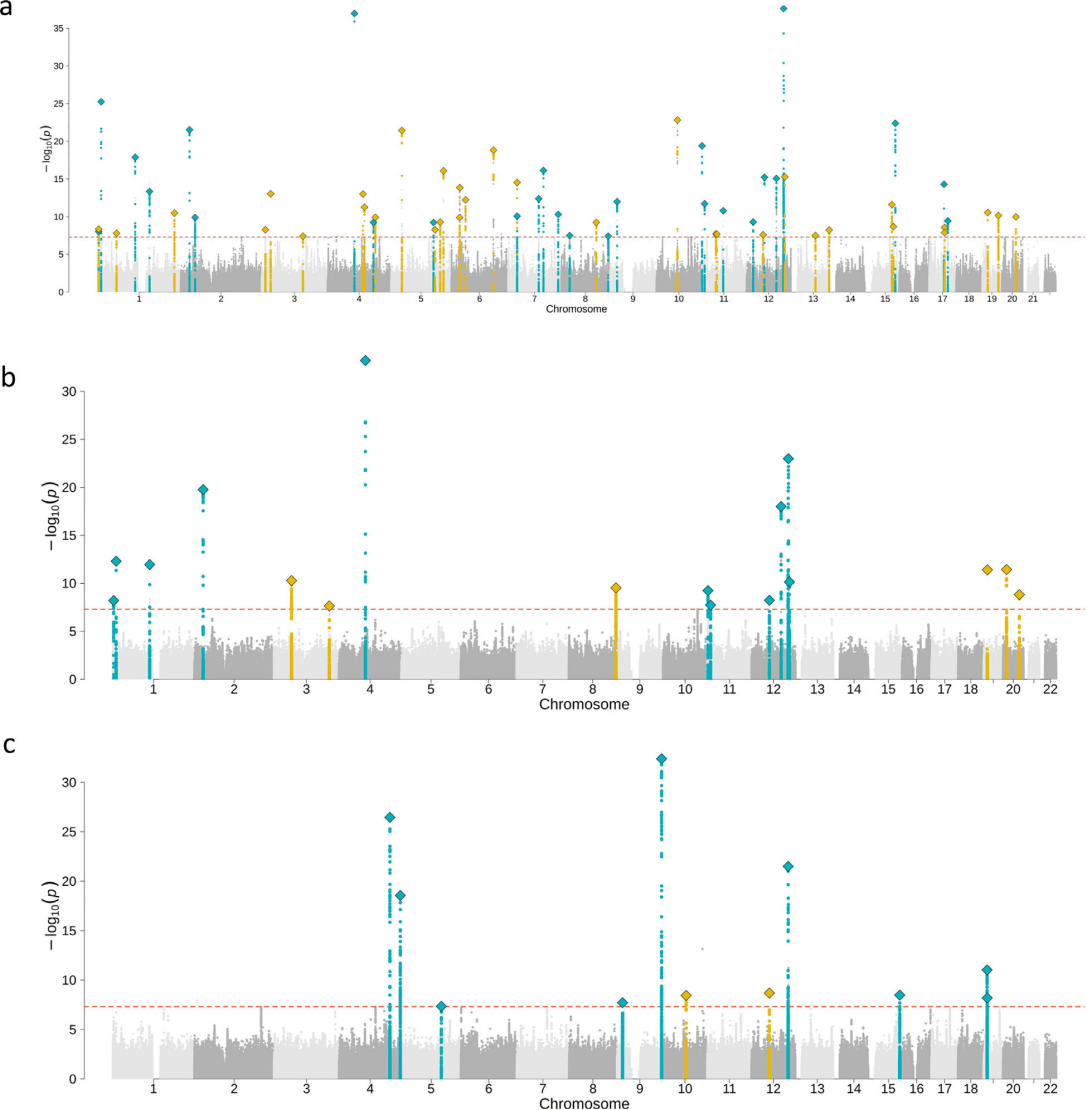
**Manhattan plot of the GWAS meta-analysis.** (a) Multi-trait meta-analysis between stroke and hypertension in Europeans. (b) Multi-trait meta-analysis between stroke and hypertension in East Asians. (c) Multi-trait meta-analysis between ischemic stroke and venous thromboembolism in Europeans. Newly identified Loci are depicted in yellow, and previously reported Loci are depicted in blue.

**Fig. 4:**
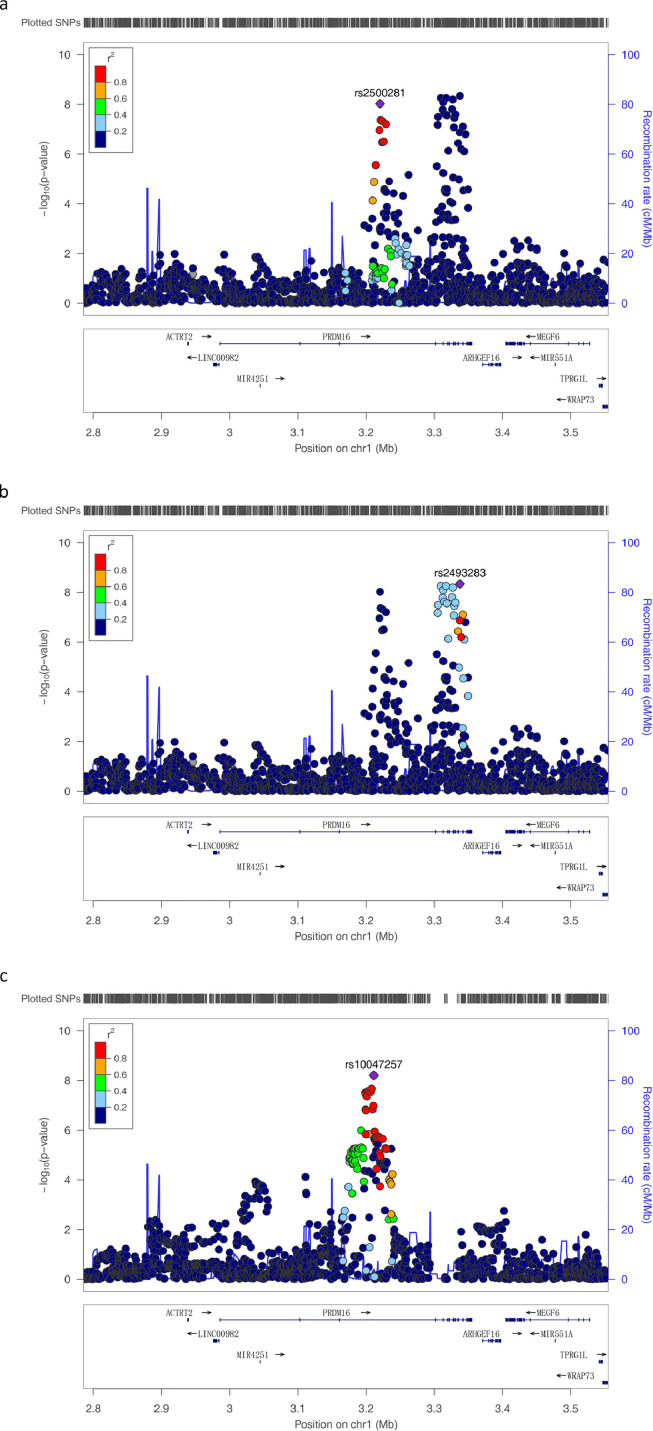
**The regional plots of the *PRDM16* locus associated with hypertension using LocusZoom.** (a) Known locus rs2500281 in Europeans. (b) Novel locus rs2493283 in Europeans. (c) Known locus rs10047257 in East Asians. Purple diamond indicates the lead SNP, and circles represent the other SNPs in the region, with coloring from the linkage disequilibrium (r^2^, based on the 1000 Genomes Project Europeans/Asians) between each SNP and the lead SNP.

The evidence obtained from the genetic correlation analysis motivated us to delve deeper into identifying potential pleiotropic loci shared between stroke and hematological risk factors. To accomplish this, we performed multi-trait meta-analysis on each genetically correlated trait pair using MTAG, and prioritized SNPs reaching genome-wide significance (*p* < 5 × 10^−8)^. During the analysis of stroke and hypertension, we were able to detect a total of 59 significant signals from individuals of European descent. Among these signals, 26 were located in loci that had been previously reported in stroke, while the other 33 loci were newly identified (Fig. 3a and Table S6). Out of these 33 loci, 29 were known in hypertension. The remaining four pleiotropic loci (Table S6) were found to be previously unreported in both stroke and hypertension. Notably, one of the four pleiotropic loci, rs2493283, is located within the gene *PRDM16*, which has been previously associated with stroke. We further utilized LocusZoom [26] to visualize the genetic associations within this specific region. As shown in Fig. 4, regional plots of association results indicated two independent signals in gene region of *PRDM16* (Fig. 4a&b). The known locus rs2500281 was found to be located in the intronic region between the third and fourth exon of *PRDM16*, while the novel locus rs2493283 was situated close to the 3′end of the gene. In our analysis of East Asians, 17 shared loci were identified from stroke and hypertension (Fig. 3b and Table S12). Among them, six loci were previously detected in hypertension, while the remaining 11 loci had previously been reported in association with stroke, including the gene region of *PRDM16*. Unlike in Europeans, regional plots indicated that only one signal (rs10047257) located within *PRDM16* (Fig. 4c) for East Asians.

**Fig. 5:**
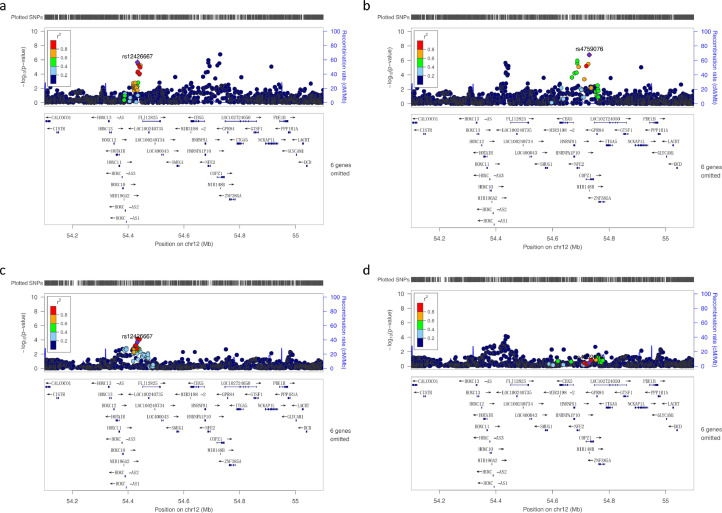
**Regional plots of the 12q13.13 locus associated with ischemic stroke using LocusZoom.** (a) Known locus rs12426667 in Europeans. (b) Novel locus rs4759076 in Europeans. (c) Locus rs12426667 in East Asians. (d) Locus rs4759076 in East Asians. Purple diamond indicates the lead SNP, and circles represent the other SNPs in the region, with coloring from the linkage disequilibrium (r^2^, based on the 1000 Genomes Project Europeans/Asians) between each SNP and the lead SNP.

**Fig. 6:**
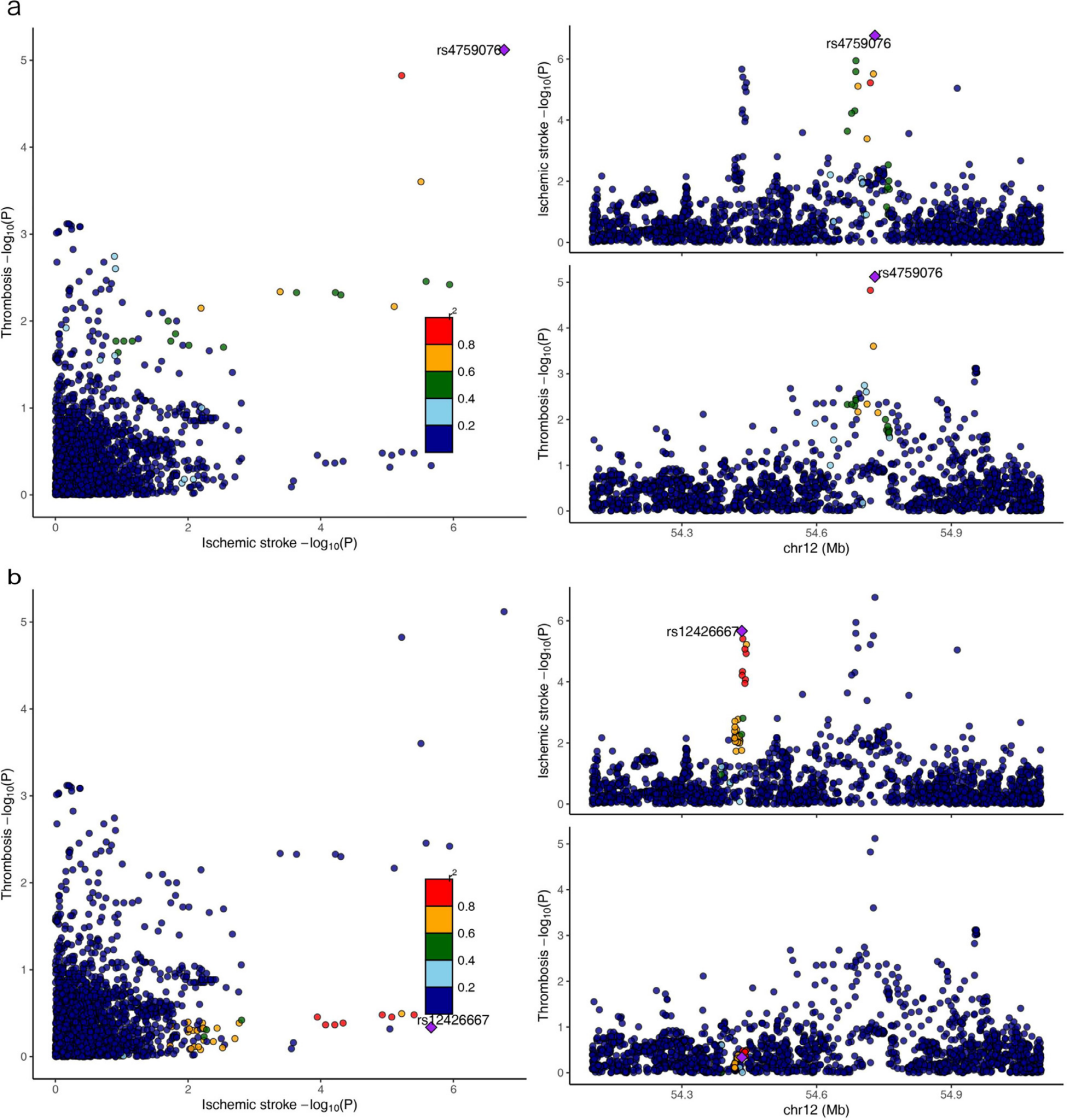
**Co-localization between ischemic stroke and venous thromboembolism in Europeans at 12q13.13 locus.** Left panel showed the merged association plot for ischemic stroke and venous thromboembolism, and right panel showed the regional plots for ischemic stroke and venous thromboembolism respectively. (a) Points are colored by LD with respect to rs4759076, which is labeled with a purple diamond. (b) Points are colored by LD with respect to rs12426667, which is labeled with a purple diamond.

We next performed MTAG analysis to identify variants affecting both stroke and venous thromboembolism in Europeans. A total of six significant loci were detected to be shared between stroke and venous thromboembolism, but all of them have been previously reported in conventional GWAS of stroke (Table S7). However, when analyzing ischemic stroke and venous thromboembolism specifically, we identified two novel loci at 10q22.1 (rs12242391) and 12q13.13 (rs4759076) (Fig. 3c and Table S7). The 10q22.1 locus (rs12242391) near gene *TSPAN15* is already known to be associated with venous thromboembolism. The rs4759076 variant, located in 12q13.13, is an intronic variant of the *COPZ1* gene, which has not been reported in relation to ischemic stroke or venous thromboembolism before. Interestingly, the *HOXC* gene cluster, a known stroke locus, is also situated in 12q13.13 but not identified by the MTAG analysis for ischemic stroke and venous thromboembolism. To further investigate this genomic region, we generated regional plots (Fig. 5a&b) and confirmed the presence of two independent signals for ischemic stroke (*HOXC* rs12426667 and *COPZ1* rs4759076) in individuals of European descent. Subsequently, we employed LocusCompare [27] to visualize co-localization of association signals in 12q13.13 (Fig. 6). Our findings revealed that co-localization between ischemic stroke and venous thromboembolism was specifically observed in the *COPZ1* gene region (rs4759076). Additionally, when examining regional plots of 12q13.13 in East Asians, only the signal surrounding *HOXC* gene cluster (rs12426667) was observed (Fig. 5c&d), suggesting that the *HOXC* gene cluster signal (rs12426667) is shared among populations of European and East Asian descent, whereas the signal from *COPZ1* region (rs4759076) is specific to Europeans. Moreover, GTEx data indicated that rs4759076 is an eQTL of *COPZ1* in thyroid (*p* = 0.00014) only. However, the eQTLGen Consortium data (Phase I) consisting of 31,684 blood samples [28] indicated that genotypes of rs4759076 are significantly correlated with multiple nearby genes (Table S17). Intriguingly, the strongest cis-eQTL effect was detected between rs4759076 and *NFE2* (*p* = 1.11 × 10^−59^). The product of *NFE2* is essential for regulating megakaryocyte biogenesis and platelet production [29]. In consistent with this, the association between rs4759076 and mean platelet volume is highly significant (*p* = 2.2 × 10^−308^) [30]. Accumulating evidence indicated that mean platelet volume is associated with mortality in acute ischemic stroke patients and may be used as a prognostic marker [31,32]. Moreover, multiple studies demonstrated that mutations of *NFE2* may cause thrombocytopenia [[33], [34], [35]], a condition characterized by abnormally low levels of platelets. Thrombocytopenia also appears to be associated with ischemic stroke [[35], [36], [37], [38]]. Besides, *NFE2L2*, an important paralog of *NFE2*, has been pointed as a potential pharmacological target for ischemic stroke [39]. Oxidative stress plays a significant role in the pathological process of stroke, characterized by the excessive generation of reactive oxygen species (ROS). The overproduction of ROS leads to brain ischemia/reperfusion injury. *NFE2L2* encodes nuclear factor-E2-related factor 2 (Nrf2), which serves as a critical regulator that stimulates the transcription of numerous antioxidant genes. Recent evidence suggests that activating Nrf2 and its target genes may offer protective effects against ischemia/reperfusion injury in the brain. Therapeutic interventions aimed at enhancing Nrf2 activity have shown promise in mitigating brain injury following stroke by alleviating oxidative stress [[39], [40], [41]]. Taken together, *NFE2* appears to be a promising gene target of stroke that warrants future study.

### 3.6 Multi-trait meta-analysis of stroke and traits of smoking and alcohol consumption

In addition to hematological risk factors, we also investigated traits related to smoking and alcohol consumption, which were also considered as risk factors of stroke. Our LDSC analysis confirmed that stroke was genetically correlated with smoking and alcohol consumption. In European populations, stroke was significantly correlated with multiple smoking traits including age of smoking initiation, smoking initiation, smoking cessation and smoking status (Table S5). Interestingly, stroke was significantly correlated with the overall alcohol intake (alcohol consumption), but not with the overall alcohol frequency (drinks per month) as shown in Table S5. In East Asian populations, stroke was also significantly correlated with age of smoking initiation and alcohol consumption (Table S5). In addition, local genetic correlation analysis using ρ-HESS revealed a significant correlation between stroke and alcohol consumption at 12q24 in East Asians (Fig. S4). We next performed MTAG analysis of stroke and the correlated traits of smoking and alcohol consumption (Table S9–11 & Table S14–15). In the analysis of Europeans, we identified four pleiotropic loci between stroke and alcohol consumption. Among them, two loci at 5q32 (rs55799797) and 11p11.2 (rs10769282) are novel in both stroke and alcohol consumption (Table S11). We also detected seven significant loci shared between stroke and smoking initiation, but all of them were previously reported in stroke (Table S10). Similarly, four pleiotropic loci were detected from stroke and smoking status, but three of them were reported in stroke and the other locus was known in smoking status (Table S9). In the analysis of East Asians, a strong pleiotropic signal between stroke and alcohol consumption was found at 12q24 (Table S15), which is highly consistent with the results from our local genetic correlation analysis.

### 3.7 Colocalization and gene-based analysis

For each pleiotropic locus identified in MTAG, Coloc was used to investigate Bayesian colocalization between stroke and corresponding hematological traits or risk factors. In the colocalization analyses between stroke and hypertension in Europeans, 10 of 33 newly identified loci in stroke were significantly colocalized (PP.H4 > 0.8) (Table S6). In East Asians, Stroke showed colocalizations with hypertension in 8 loci, and 2 loci were found to be previously unreported in stroke (Table S12). SMR was further performed to prioritize candidate stroke causal genes associated with pleiotropic loci identified in MTAG. By integrating the European-ancestry MTAG statistics and cis-eQTL summary data of whole blood from the GTEx, we identified multiple gene expression-trait associations (Supplementary Tables 6–11).

## 4 Discussion

In present study, we initially assessed the genetic correlation of stroke and its subtypes across and within different ancestries. Despite the absence of a significant cross-ancestry genetic correlation for any stroke subtype, strong positive correlations between stroke and its subtypes are evident between European and East Asian populations (Table S3). This insignificant cross-ancestry genetic correlation may be attributed to small sample sizes. The limited number of stroke cases among Asians reduces statistical power, impeding the identification of meaningful genetic associations. Especially in the GWAS of cardioembolic stroke, with a relatively small sample size of only 926 cases, no significant association was found between cardioembolic stroke and any other stroke type in the genetic correlation analysis in East Asians (Fig. S1 b). Subsequently, we further elucidate the shared genetic architecture between stroke and potentially related hematological traits or risk factors in European and East Asian populations, respectively. Stroke subtypes among Europeans exhibit broader associations with blood pressure-related traits and smoking behavior compared to East Asians, possibly due to the larger sample size and higher statistical power within the European dataset. Interestingly, large artery stroke was negatively correlated with hemoglobin concentration in East Asians, which was not observed in Europeans. This finding could potentially be attributed to variances in lifestyle habits and genetic backgrounds across different populations.

The recent published GWAS of stroke launched by the GIGASTROKE consortium has collected genetic data of 110,182 patients with stroke and 1,503,898 control individuals from multiple ancestries [7]. The above stroke GWAS employed three meta-analysis method including the conventional IVW (inverse-variance weighted) meta-analysis, MR-MEGA (meta-regression of multi-ethnic genetic association) [42] and MTAG, leading to the identification of 89 significant loci. Among them, 26 loci were detected using MTAG with WMH (white matter hyperintensity volume), CAD (coronary artery disease) and atrial fibrillation [7]. In addition to the above stroke associated traits, we found that a substantial number of known stroke loci are also present in hypertension (Table S16). Considering hypertension as a critical risk factor for stroke, we performed a more in-depth MTAG analysis to exploring the shared genetic mechanism between these two conditions.

A strong genetic correlation between stroke and hypertension was observed in both Europeans and East Asians. Furthermore, we identified a significant local genetic correlation at 12q24, a region previously associated with both conditions. To gain further insights, we employed MTAG analysis to identify shared loci between stroke and hypertension. According to the average χ2 test statistic, MTAG led to an increase in the effective sample size, specifically from 1,308,460 to 1,981,407 in Europeans and from 264,655 to 347,679 in East Asians. With the enhanced statistical power, we successfully identified 59 pleiotropic loci associated with both stroke and hypertension in Europeans, and 17 such loci in East Asians. Importantly, four of these loci were novel and had not been previously linked to either condition. Notably, rs2493283, one of the novel pleiotropic loci, was found within the genomic region associated with stroke encompassing *PRDM16*. Subsequent detailed investigations confirmed that rs2493283 operated independently of the known locus rs2500281 in *PRDM16*, implying a complex genetic mechanism at play in this specific gene region concerning stroke. Another locus among the four novel pleiotropic ones, rs1028958, was situated near the gene *KLF5*, which encodes a zinc-finger transcription factor with involvement in various biological processes. Multiple lines of evidence further substantiated that *KLF5* exerts a regulatory role in both stroke and hypertension [[43], [44], [45]]. Additionally, we conducted an MTAG analysis between stroke and blood pressure measurements, and the results largely corroborated the findings pertaining to stroke and hypertension (Table S8 & S13).

We next delved into the investigation of pleiotropic loci between stroke and coagulation disorders or coagulation factors, as a few potential therapeutic targets identified in stroke GWAS, like *F11* and *PROC*, play roles in the coagulation mechanism and related disorders. However, no significant locus was identified through the MTAG of stroke and coagulation factors. The MTAG analysis of stroke and venous thromboembolism, with a slightly increased effective sample size from 1,308,460 to 1,420,136, identified six significant loci, which were already reported in stroke. Interestingly, we discovered two novel loci, rs12242391 at 10q22.1 and rs4759076 at 12q13.13, in the MTAG analysis of ischemic stroke and venous thromboembolism. The 10q22.1 locus (rs12242391) near gene *TSPAN15* was previously reported in venous thromboembolism. According to GTEx, variant rs12242391 is an eQTL of *TSPAN15* in multiple tissues including whole blood (*p* = 2.1 × 10^−11^). While the biological link between *TSPAN15* and venous thromboembolism is still obscure, our results suggest that *TSPAN15* might contribute to the pathology of both ischemic stroke and venous thromboembolism through a similar mechanism. Of note, we also identified a novel locus (rs4759076) located in the *COPZ1* gene, which has never been reported in ischemic stroke or venous thromboembolism before. Interestingly, our eQTL analysis suggested the target gene is *NFE2* as detailed in the results section. The exploration of *NFE2* and related mechanisms could pave the way for novel therapeutic strategies for ischemic stroke treatment.

We further investigated two risk factors associated with stroke, smoking and alcohol consumption. In East Asians, our local genetic correlation analysis revealed a significant link between stroke and alcohol consumption at 12q24. Subsequently, MTAG analysis identified a single significant pleiotropic locus (rs77753011) related to both stroke and alcohol consumption at this specific region (Table S15). Notably, the rs77753011 variant demonstrated linkage disequilibrium (LD) with a missense variant of *ALDH2* (rs671, *R^2^* = 0.68) exclusively in the East Asian population. However, when examining the original summary statistics of alcohol dependence, we found that the standard error (SE) for rs671 was zero, resulting in an undefined Z-score (BETA/SE). Consequently, rs671 was excluded from the MTAG analysis. Similarly, the MTAG analysis for stroke and smoking behaviors (cigarettes per day) identified only one significant pleiotropic locus at 12q24. The top SNP at this locus, rs79105258 (Table S14), was also in LD with rs671 (*R^2^* = 0.57), but this variant was missing in the original summary statistics of the smoking behaviors GWAS and thus excluded from the analysis. This suggests that the 12q24 locus detected by MTAG analysis for stroke and alcohol consumption, as well as for stroke and smoking behaviors, is likely influenced by rs671 in East Asians. The *ALDH2* rs671 variant leads to a loss of ALDH2 enzymatic activity, resulting in adverse reactions to acetaldehyde, such as flushing, headaches, and nausea, which effectively reduces alcohol consumption [46]. Consequently, this genetic variant confers a protective effect against stroke. However, various studies have indicated that individuals carrying this variant who still consume alcohol face an increased susceptibility to stroke, likely due to higher concentrations of acetaldehyde after drinking [47,48]. As a result, this genetic variant leads to diverse alcohol consumption patterns and varying susceptibility to stroke. In European populations, we identified four pleiotropic loci between stroke and alcohol consumption. Among them, two loci at 5q32 (rs55799797) and 11p11.2 (rs10769282) are novel in both stroke and alcohol consumption. It is worth mentioning that we found four pleiotropic loci between small vessel stroke and alcohol consumption including the 4q23 (rs1789896) locus. The lead variant rs1789896 is an eQTL of *ADH1C*, which encodes class I alcohol dehydrogenase. This variant may influence alcohol consumption and further confer risk to small vessel stroke in Europeans.

In this study, we conducted an in-depth multi-trait analysis on stroke and hematological traits or risk factors. The increased statistical power enabled us to identify more than thirty novel loci through joint analysis of stroke and hypertension, providing a comprehensive understanding of the pleiotropic genetic architecture of both conditions. As hypertension is a crucial risk factor for stroke, many loci may exhibit vertical pleiotropy, arising from the causal relationship between these two traits. However, it is also essential to consider that certain genetic loci might demonstrate horizontal pleiotropy, directly influencing both traits. Further functional research is required to explore causal variants and underlying mechanisms at these loci, offering deeper insights into the etiology of stroke. Furthermore, we extended our investigation to smoking and alcohol consumption, two additional risk factors for stroke. Our analysis revealed that the identified variants contribute to stroke risk by regulating key enzymes involved in alcohol metabolism, demonstrating vertical pleiotropy. Importantly, we made a novel discovery during our analysis of ischemic stroke and venous thromboembolism. We provided compelling evidence indicating that the target gene *NFE2* may contribute to the risk of ischemic stroke through its role in regulating platelet production.

## 5 Conclusion

In summary, our study sheds new light on the shared genetic mechanisms between stroke and hematological traits or risk factors. These findings have the potential to open up new avenues for the prevention and treatment of stroke, offering valuable insights into its genetic basis and associated risk factors.

## Declaration of competing interest

The authors declare that they have no conflicts of interest in this work.

## Appendix Supplementary materials

Image, application 1

Image, application 2
