# Supplementary material for: Multi-trait genetic analysis identifies novel pleiotropic loci for stroke and hematological traits or risk factors
Source: Fundam Res. 2024 May 20;6(4):2701–10. doi: 10.1016/j.fmre.2024.05.004 (PMC13424371; doi:10.1016/j.fmre.2024.05.004)
Supplement: Supplementary file 1 [file mmc1.docx]

Supplementary Fig.1. Heatmaps of pairwise genome-wide genetic correlations between stroke and subtypes in Europeans (a) and East Asians (b). The matrix displays the strength of correlation varying in shade by magnitude of correlation and significant associations are marked with asterisks (*, *p* < 0.05; **, *p* < 0.01; ***, *p* < 0.001).


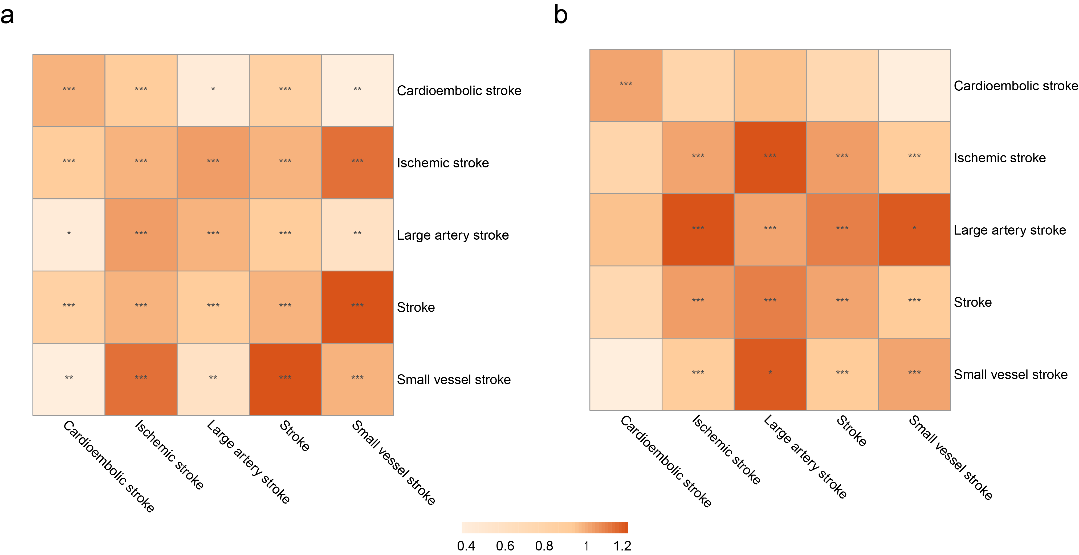


Supplementary Fig. 2. Heat maps showing patterns of cell-type-specific enrichments of SNP-heritability for genetically associated traits across 88 cell types- or tissues-specific annotations. Each checkered rectangle reflects the z-score, scaled by traits. Red indicates enrichment, blue indicates depletion. Deeper color represents stronger magnitude of effects. Asterisks represent statistical significance. The category of cell-types is color coded to the left. (a) DNase (DNase I hypersensitive sites), (b) H3K27ac, (c) H3K36me3, (d) H3K4me1, (e) H3K4me3, and (f) H3K9ac.Thromb, Venous thromboembolism; CS, Cardioembolic stroke; LAS, Large artery stroke; SVS, Small vessel stroke; CS, Cardioembolic stroke; IS, Ischemic stroke; S, Stroke; HTN, Hypertension.

**a** DNase


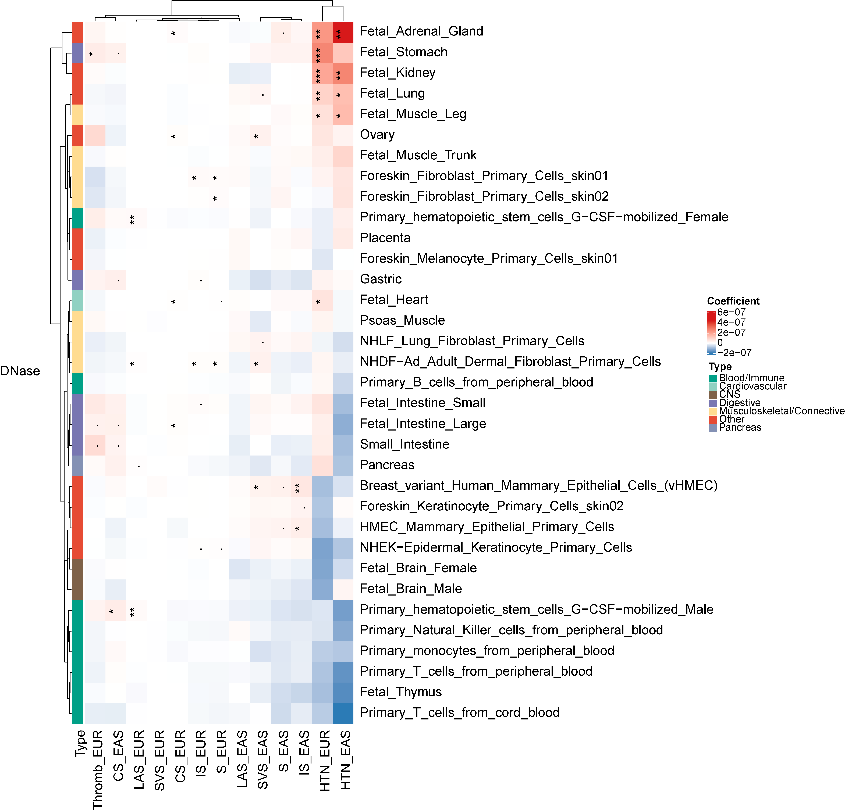


**b** H3K27ac


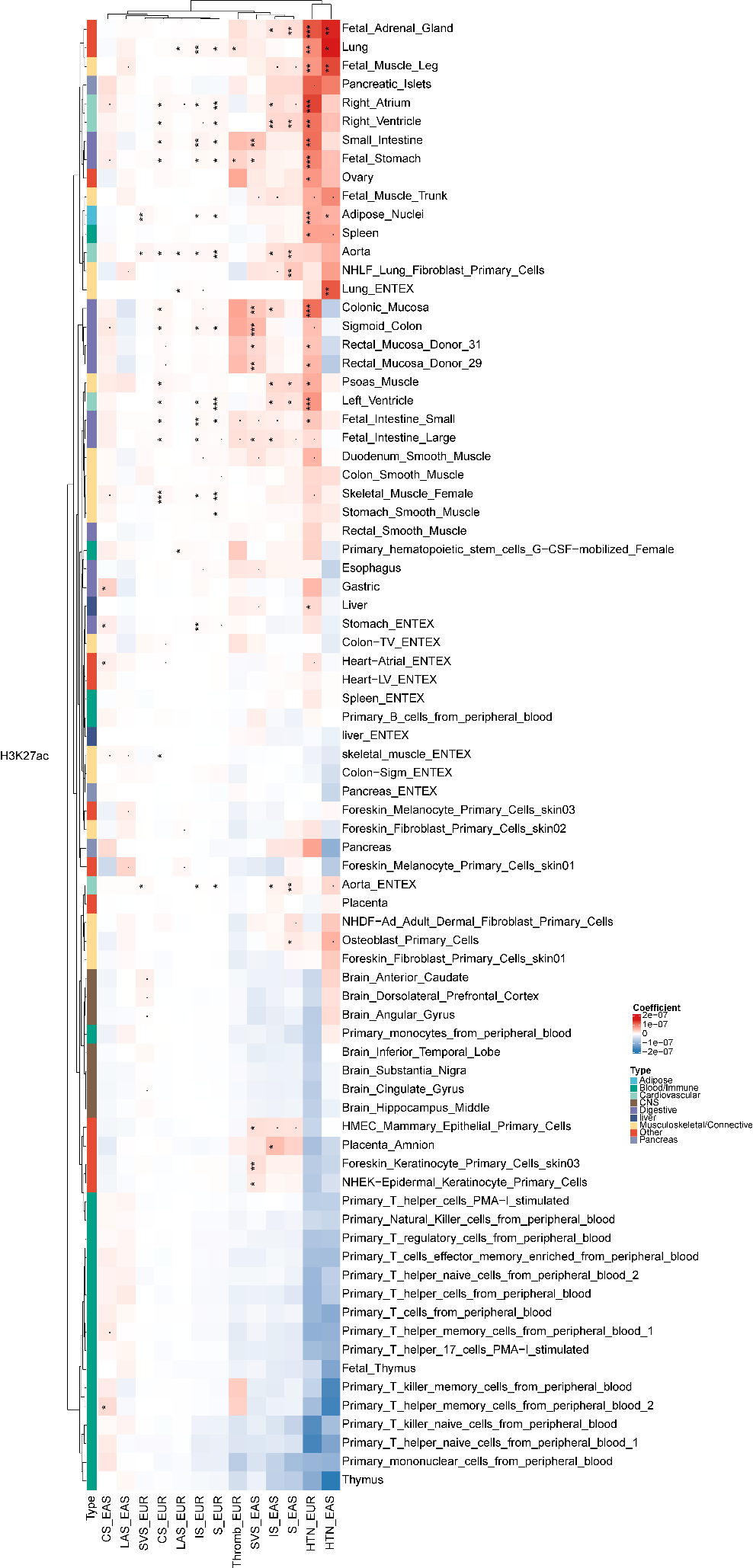


**c** H3K36me3


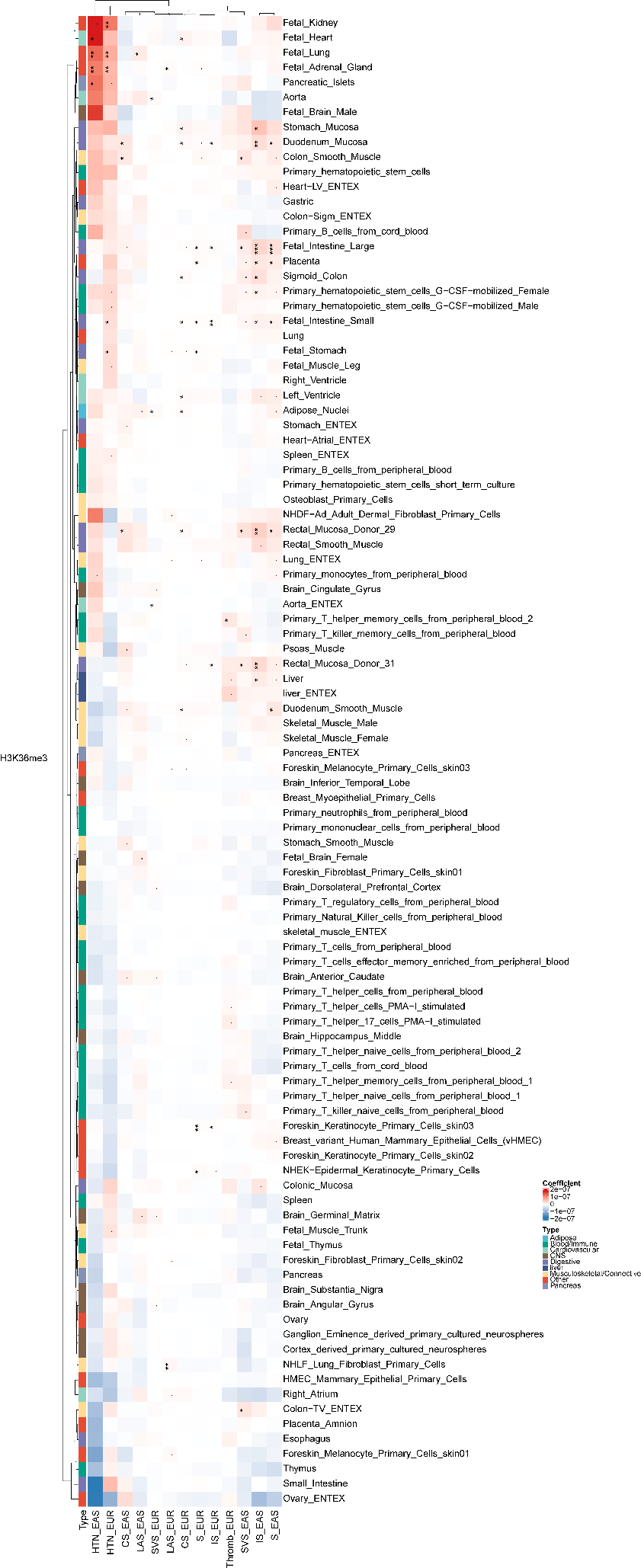


**d** H3K4me1


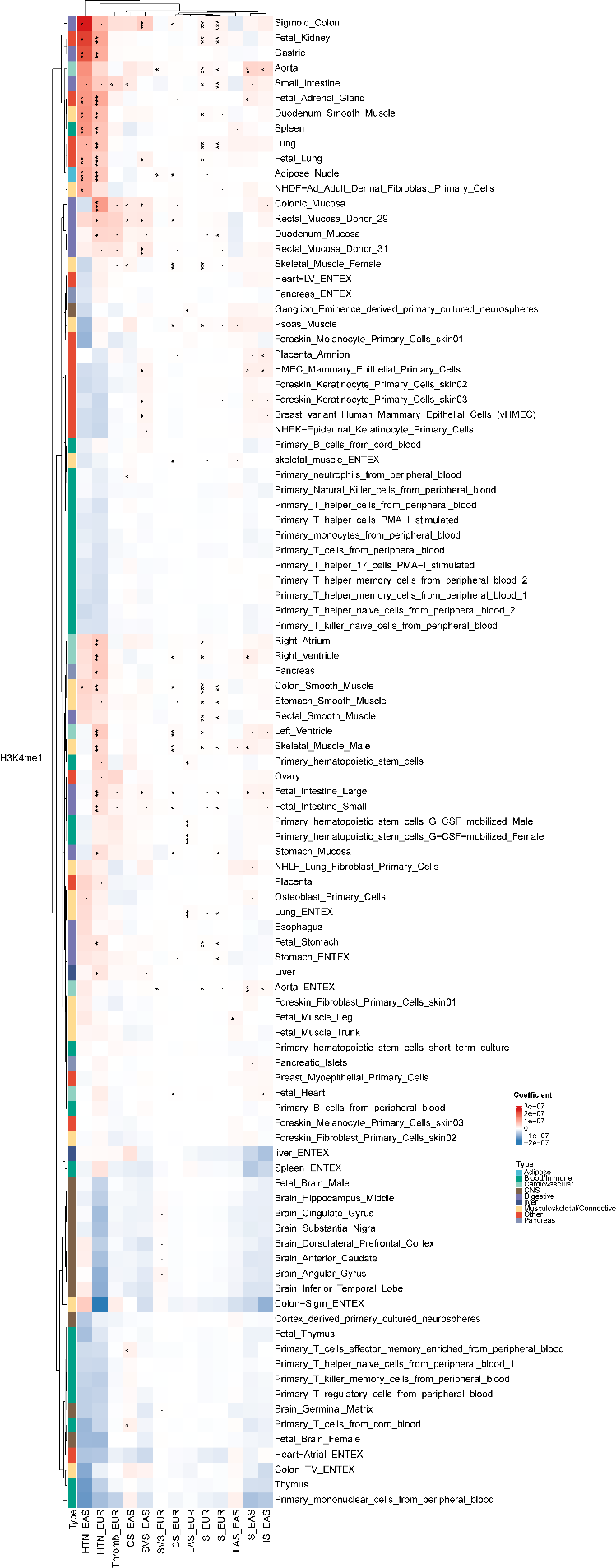


**e** H3K4me3


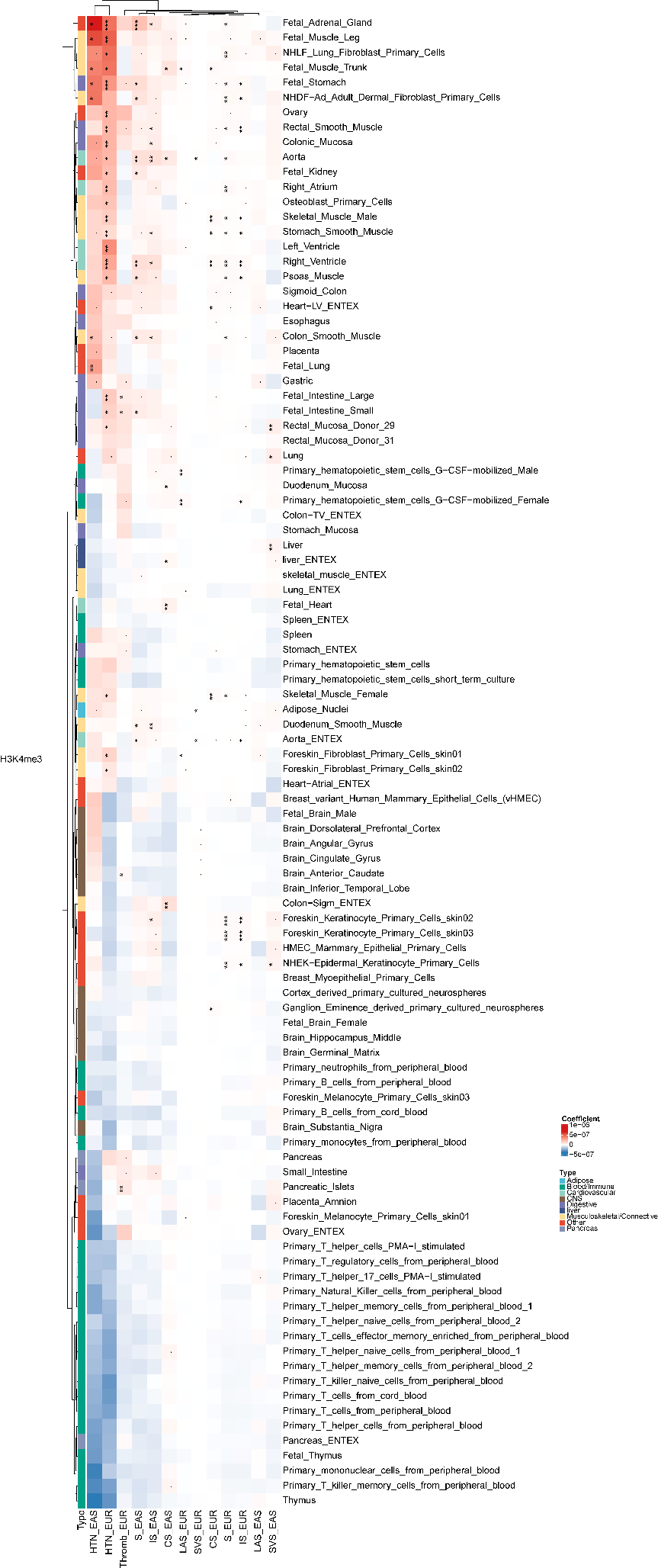


**f** H3K9ac


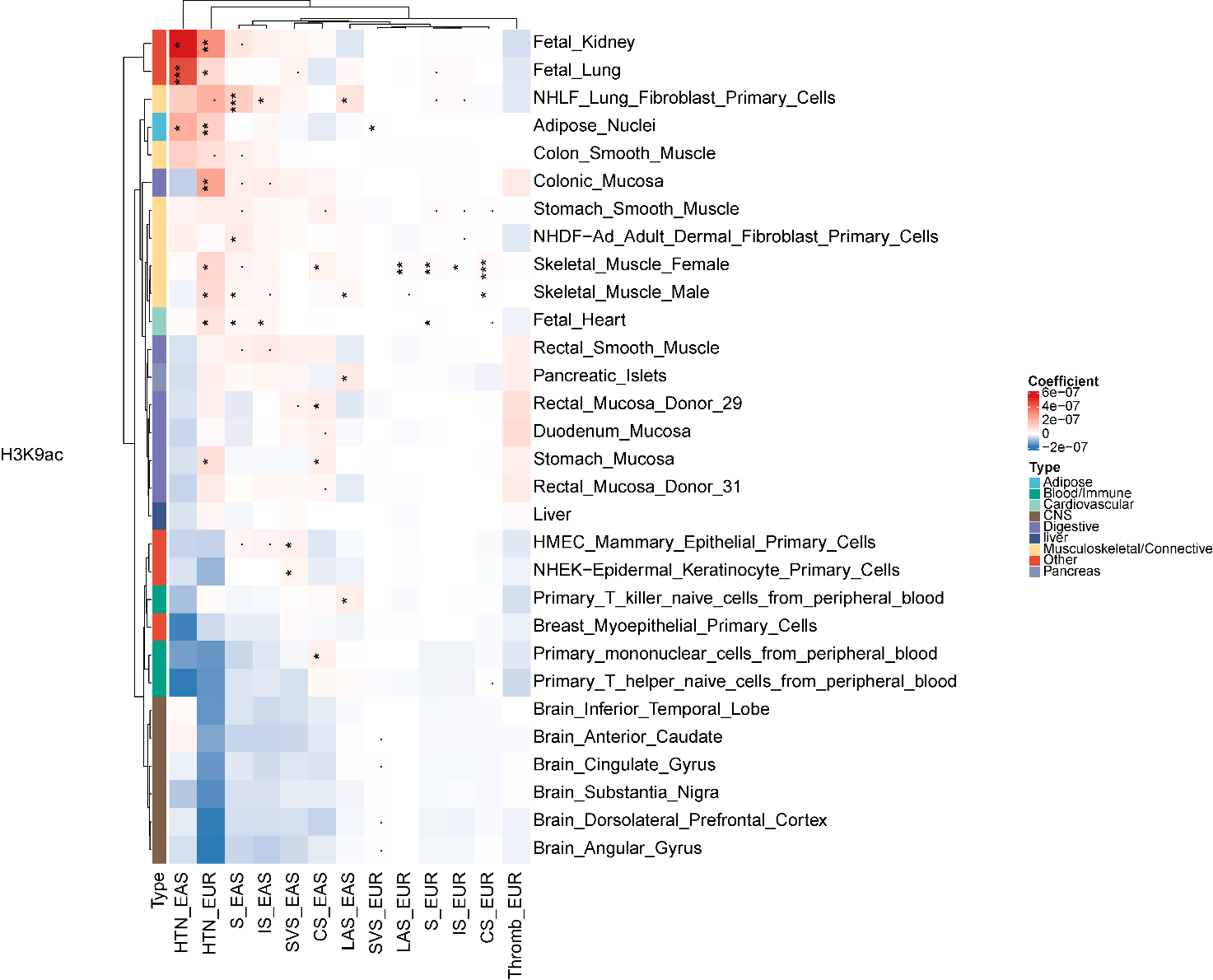


Supplementary Fig. 3. Local genetic correlation between stroke and blood pressure measurements in East Asians. (a) Manhattan plot showing the estimates of local genetic correlation, genetic covariance, and SNP heritability between stroke and hypertension in East Asians. (b) Manhattan plot showing the estimates of local genetic correlation, genetic covariance, and SNP heritability between stroke and diastolic blood pressure (DBP) in East Asians. (c) Manhattan plot showing the estimates of local genetic correlation, genetic covariance, and SNP heritability between stroke and systolic blood pressure (SBP) in East Asians. Red bars represent loci showing significant local genetic correlation after multiple testing adjustment (*p* < 0.05/1,703).

**a**

**b**

**c**

Supplementary Fig. 4. Local genetic correlation between stroke and alcohol consumption in East Asians. Manhattan plot showing the estimates of local genetic correlation, genetic covariance, and SNP heritability between stroke and alcohol consumption in East Asians. Red bars represent loci showing significant local genetic correlation after multiple testing adjustment (*p* < 0.05/1,703). ALC, alcohol consumption.
